# Impact of phosphorus supply and arbuscular mycorrhizal fungi inoculation on rhizosphere fungal communities and growth of *Macadamia integrifolia* seedlings

**DOI:** 10.3389/fpls.2026.1788218

**Published:** 2026-04-10

**Authors:** Ya Ning, Zhonghua Wu, Yuchun Chen, Tingmei Yang, Xiyong He, Lichen Feng, Hong Zhao, Hai Yue

**Affiliations:** 1 Yunnan Institute of Tropical Crops, Jinghong, Yunnan, China; 2 Yunnan Provincial Woody Oil Technology Innovation Center, Jinghong, Yunnan, China; 3 Yunnan Provincial Macadamia Agricultural Engineering Research Center, Jinghong, Yunnan, China

**Keywords:** arbuscular mycorrhizal fungi, phosphorus supply, *Macadamia integrifolia*, rhizosphere fungal community, plant growth, phosphorus allocation

## Abstract

**Introduction:**

Phosphorus (P) limitation constrains the growth of *Macadamia integrifolia* in subtropical soils, despite intrinsic adaptations such as cluster roots and rhizosphere P mobilization. However, the role of arbuscular mycorrhizal fungi (AMF) in enhancing P acquisition under varying P availability and inoculum identity remains unclear.

**Methods:**

We conducted a controlled pot experiment with three P levels (P0, P1, P2) and two AMF treatments (indigenous consortium and *Glomus mosseae*), assessing seedling growth, biomass allocation, organ-level P concentrations, rhizosphere soil properties, and fungal community composition.

**Results:**

Seedling growth and biomass allocation responded non-linearly to P, with maximal aboveground biomass at P1. Notably, *G. mosseae* inoculation under P1 increased aboveground biomass by 42.9% and root P concentration by 18.4%, whereas the indigenous AMF consortium enhanced leaf P and rhizosphere P under low-P conditions. AMF colonization was strongly associated with total plant biomass but decoupled from short-term P uptake efficiency. Rhizosphere fungal α-diversity and community composition varied with P supply and AMF identity, with Glomeromycota enrichment and reduced saprotroph abundance in inoculated treatments.

**Discussion:**

These findings demonstrate context-dependent AMF effects that integrate strain-specific functional traits with intrinsic plant P-acquisition strategies, highlighting functional differentiation between growth promotion and immediate nutrient acquisition. This study provides quantitative guidance for optimizing growth and P-use efficiency in low-P-adapted woody crops.

## 1 Introduction

Phosphorus (P) limitation is a major constraint for crop production in highly weathered subtropical soils, where P is often immobilized through precipitation and strong sorption processes (Penn and Camberato, 2019). Improving phosphorus-use efficiency through plant–rhizosphere interactions is therefore a key objective in sustainable nutrient management (Andrade et al., 2022; Jacoby et al., 2017).

Macadamia integrifolia, a high-value woody crop native to nutrient-depleted Australian soils, exhibits adaptive traits associated with low-P environments (Pan et al., 2025). As a member of the Proteaceae, it is capable of modifying the rhizosphere to mobilize poorly soluble P pools (Vance et al., 2003). These adaptations raise a fundamental ecological question: does arbuscular mycorrhizal fungal (AMF) symbiosis provide additional phosphorus-acquisition benefits in plant species already adapted to low-P conditions, or is its function largely redundant?

Arbuscular mycorrhizal fungi are well-documented for their capacity to enhance plant P uptake by extending the nutrient-absorbing surface area beyond the depletion zone and by accessing organic P sources through hyphal networks (Richardson et al., 2009; Shukla et al., 2025; Wang et al., 2023). Nevertheless, accumulating evidence suggests that AMF effects are highly context dependent and may vary with nutrient availability and inoculum identity (Jiang et al., 2021; Kobae, 2019; Zhang et al., 2018). In crops possessing intrinsic P-acquisition mechanisms, the net outcome of AMF symbiosis may reflect a dynamic trade-off between carbon investment and P return, modulated by environmental P supply (Rodríguez-Caballero et al., 2017).

A key but understudied aspect is the environmental adaptability contrast between indigenous AMF consortia and introduced single-species inocula across phosphorus gradients (Ilyas et al., 2024). Indigenous AMF consortia, shaped by long-term co-evolution with local soil conditions and host genotypes, may exhibit adaptive traits such as low carbon cost or high stress tolerance under nutrient limitation (Gazey et al., 2004). Despite its ecological and practical relevance, this contrast has rarely been examined in low-P-adapted woody crops through factorial experiments that simultaneously assess plant performance, phosphorus allocation, and rhizosphere fungal community responses.

To address this knowledge gap, we conducted a controlled factorial experiment combining three phosphorus supply levels with two AMF inoculation treatments (an indigenous AMF consortium and Glomus mosseae) to investigate how nutrient background and inoculum identity jointly influence plant growth, phosphorus allocation, and rhizosphere fungal community structure in Macadamia integrifolia seedlings. We hypothesized that (i) plant growth and P allocation would exhibit non-linear responses across P levels; (ii) indigenous AMF would show stronger associations under low-P conditions; and (iii) introduced AMF would exert more pronounced effects under moderate phosphorus supply.

## 2 Materials and methods

### 2.1 Study site and experimental conditions

The pot experiment was conducted from October 2023 to November 2024 in the greenhouse of the Yunnan Institute of Tropical Crops, China (22.05° N, 100.80° E; 565 m a.s.l.). The region experiences an annual mean temperature of 22.6 °C, 86% relative humidity, and approximately 1500 mm of annual rainfall. Greenhouse conditions followed standard macadamia management practices. Irrigation was applied as needed, and no additional fertilizers were added during the experiment.

The growth substrate was brick-red raw soil collected from a macadamia orchard at a depth of 5–20 cm. The orchard has been planted with macadamia for over 20 years. Its main physicochemical properties were as follows: total nitrogen 0.74 g kg^-^¹, available phosphorus 0.32 mg kg^-^¹, available potassium 80.27 mg kg^-^¹, pH 4.68, and organic matter 5.4 g kg^-^¹. The substrate was air-dried, cleaned of debris, sieved (2 mm), and sterilized by autoclaving at 121 °C and 121 kPa for 30 minutes prior to use. After sterilization, the soil was stored under dry conditions until pot preparation.

### 2.2 Plant material and AMF inoculum

Seeds of *Macadamia integrifolia* (cv. H2) were used in the study Uniform, healthy kernels were surface-sterilized in 0.5% NaClO for 10 minutes, rinsed thoroughly with sterile water, and germinated at 28°C in darkness for 7–10 days. When radicles reached 2–3 cm, uniform seedlings were transplanted.

Three AMF inoculation treatments were established:

1. CK (control): No AMF inoculum added; received sterilized inoculum carrier to account for any non-AMF effects.
2. NM (indigenous AMF consortium): Indigenous AMF inoculum was collected from the rhizosphere soil (5–20 cm depth) of a long-term macadamia orchard. The inoculum consisted of a mixture of soil, roots, and spores, with an average spore density of approximately 150 spores per 100 g soil (determined by wet sieving and sucrose centrifugation). This native density was directly used as the inoculation rate to preserve the natural AMF community structure and simulate field conditions. Dominant spore morphotypes included Ambispora, Gigaspora, and Glomus species, as identified by morphological characteristics. Molecular characterization (see Section 2.8) later confirmed the presence of multiple AMF phylotypes.
3. GM (single-species inoculum): G*lomus mosseae* strain supplied by Yangtze University (propagated on *Trifolium repens*), 600 spores 100 g^-^¹, 600 spores per 100 g soil, bacteria-free. *G. mosseae* was chosen due to its well-established role in promoting plant growth through enhanced phosphorus uptake and its effectiveness as a model AMF species in similar experiments.

### 2.3 Experimental design and AMF inoculation

A randomized complete block design with two factors was adopted: (1) AMF inoculation (CK, NM, G-M) and (2) phosphorus supply level (P0, 0 mg·kg^-^¹; P1, 40 mg·kg^-^¹; P2, 200 mg·kg^-^¹). Each of the nine treatment combinations was replicated three times, resulting in 27 experimental units. These three phosphorus levels were carefully selected to represent a gradient from deficient to excessive conditions. P0 (0 mg·kg^-^¹) served as a severe phosphorus deficiency control to establish a baseline for evaluating AMF effectiveness under stress. P1 (40 mg·kg^-^¹) was chosen to represent a moderate or agronomically adequate phosphorus supply. P2 (200 mg·kg^-^¹) was designed to represent excessive phosphorus conditions, reflecting common over-fertilization scenarios observed in local macadamia production systems. By constructing this phosphorus gradient, this study aims to systematically investigate the differential responses of AMF inoculation effects under varying soil phosphorus backgrounds.

Each treatment was replicated 3 times, ensuring sufficient biological replication for statistical analysis. Across all treatments, P fertilizer was supplied as Ca(H_2_PO_4_)_2_, a source of phosphorus in the form of calcium dihydrogen phosphate, while nitrogen, potassium, and micronutrients were supplemented with NH_4_NO_3_ (ammonium nitrate, equivalent to 52.5 mg N·kg^-^¹ soil) and KNO_3_ (potassium nitrate, equivalent to 20.9 mg N·kg^-^¹ soil) at 150 mg·kg^-^¹ each. Micronutrients included iron (Fe), manganese (Mn), zinc (Zn), and copper (Cu) in their sulfate forms (5 mg·kg^-^¹ each). These micronutrients are essential for various enzymatic processes and plant growth. Plastic pots (diameter 35 cm, height 40 cm) were disinfected with 75% ethanol (30 min) before use.

AMF inoculation followed the sandwich method. To ensure consistent application, in NM, each pot received 1000 g indigenous inoculum; in GM, each pot received 100 g G. mosseae inoculum; CK received the same amount of sterilized inoculum to equalize substrate properties. Each pot contained 8 kg sterilized substrate; P fertilizer was thoroughly mixed into the entire substrate volume according to the designated P treatment before transplanting. Seedlings were placed so that roots directly contacted the inoculum layer, and covered with moistened sterile substrate.

It should be noted that this study was designed to evaluate plant growth responses and rhizosphere community shifts at the organismal and community levels, rather than to conduct physiological characterization of AMF isolates. Therefore, strain-specific functional traits (e.g., hyphal growth kinetics) were not directly measured, and interpretations regarding AMF functional mechanisms are inferred from plant performance and community-level patterns.

### 2.4 Sampling and plant measurements

Plant height (from the soil surface to the apical meristem) and stem diameter (at 5 cm above soil surface) were measured at regular intervals throughout the experiment using a ruler and digital calipers, respectively. At the harvest in August 2024 (after approximately 44 weeks of growth), each seedling was carefully removed from the pot, and the soil was gently shaken off to expose the root system. Plants were then separated into leaves, stems, and roots.

All tissues were briefly rinsed with deionized water to remove adhering soil particles, blotted dry, and oven-dried following a standardized procedure: 105 °C for 30 min to deactivate enzymes and halt metabolic activity, followed by drying at 70 °C for 72 h until a constant weight was achieved. The dry biomass of each organ was recorded using an analytical balance (precision ±0.001 g). Dried tissues were subsequently ground into a fine powder with a stainless-steel grinder and passed through a 0.25 mm sieve for nutrient analysis.

Tissue phosphorus (P) concentration was determined by digesting 0.2 g of ground material with a mixed H_2_SO_4_–H_2_O_2_ solution under controlled heating until the digest became clear. The P content of the digest was quantified using the vanadomolybdate yellow colorimetric method, and absorbance was measured at 420 nm with a UV–Vis spectrophotometer, following the procedures of (Murphy and Riley, 1962).

### 2.5 Assessment of AMF root colonization

To verify the effectiveness of soil sterilization and to quantify AMF colonization, root subsamples were collected from each plant at harvest. Fresh fine roots were cleared in 10% (w/v) KOH at 90 °C for 30 min, rinsed with deionized water, acidified in 1% HCl for 10 min, and then stained with 0.05% trypan blue in lactoglycerol at 90 °C for 15 min. Stained roots were destained in lactoglycerol and cut into 1-cm segments. Thirty randomly selected segments per sample were mounted on slides and examined under a light microscope at 200× magnification. AMF colonization rate (%) was calculated as the percentage of root segments containing arbuscules, vesicles, or hyphae using the gridline intersect method (Mc et al., 1990). Colonization intensity was also recorded as the proportion of root length colonized.

### 2.6 Soil physicochemical analyses

Rhizosphere soil was collected by gently brushing off soil adhering to the root surface. Non-rhizosphere soil was defined as soil not directly attached to the roots. Fresh rhizosphere soil was immediately transferred to sterile 2 mL microcentrifuge tubes, kept on dry ice during transport to the laboratory, and stored at −80 °C until DNA extraction.

The remaining rhizosphere and non-rhizosphere soil fractions were air-dried, sieved through a 2 mm mesh, and used for chemical analyses. Soil pH was determined in a 1:2.5 (w/v) soil-to-deionized-water suspension using a calibrated pH meter. Soil available phosphorus was extracted using *NH_4_F–HCl* following established protocols (Bray and Kurtz, 1945; Olsen and Sommers, 1982). The soil was mixed with the extractant, shaken for a fixed period, centrifuged, and filtered prior to colorimetric measurement of available P (Murphy and Riley, 1962).

### 2.7 DNA extraction and sequencing

Total genomic DNA was extracted from rhizosphere soil samples using the E.Z.N.A.^®^ Soil DNA Kit (Omega Bio-tek, Norcross, GA, USA) according to the manufacturer’s instructions. DNA concentration and purity were assessed using a NanoDrop 2000 spectrophotometer (Thermo Scientific, USA), and DNA integrity was verified by 1% agarose gel electrophoresis. The internal transcribed spacer (ITS) region of fungal rDNA was amplified using primer pair ITS1F (5′-CTTGGTCATTTAGAGGAAGTAA-3′) and ITS4R (5′-TCCTCCGCTTATTGATATGC-3′). PCR amplification was performed on a T100 Thermal Cycler (Bio-Rad, USA) using FastPfu DNA Polymerase (TransGen Biotech, Beijing, China). Amplicons were separated on 2% agarose gels, purified using a PCR Clean-Up Kit (Axygen, USA), and quantified prior to library construction. Sequencing libraries were prepared using the NEXTFLEX^®^ Rapid DNA-Seq Kit (Bioo Scientific, USA) and sequenced on an Illumina NextSeq 2000 platform (Shanghai Majorbio, China). Equimolar amounts of purified amplicons were pooled and paired-end sequenced on an Illumina MiSeq platform (Illumina, San Diego, CA, USA) at Shanghai Majorbio Bio-Pharm Technology Co., Ltd.

The ITS region was selected to characterize overall fungal community composition in the rhizosphere. Although AMF-specific markers based on the small subunit (SSU) region (e.g., NS31/AM1) can provide higher taxonomic resolution for arbuscular mycorrhizal fungi (AMF), the objective of the present study was to evaluate broader fungal community shifts associated with AMF inoculation and phosphorus supply rather than to quantify AMF taxa exclusively.

### 2.8 Bioinformatics analysis

Raw sequencing reads were processed in QIIME2 for quality control, including the removal of adapters and low-quality bases. The quality filtering statistics for each sample, including read counts, Q20/Q30 values, and effective rates, are summarized in **Supplementary Table S1**. Denoising, error correction, and chimera removal were performed using the DADA2 pipeline to generate high-resolution amplicon sequence variants (ASVs). The rarefied ASV table was used to calculate α-diversity indices (Chao1 richness, Shannon diversity, and Simpson evenness), and pairwise differences among treatments were assessed with Wilcoxon rank-sum tests. β-Diversity was evaluated using Bray–Curtis distance matrices and visualized by principal coordinates analysis (PCoA). Differences in community composition were tested with PERMANOVA (999 permutations). Taxonomic assignment of ASVs was carried out against the UNITE reference database using a naïve Bayes classifier. Fungal functional guilds were inferred with FUNGuild, retaining only assignments with high or moderate confidence to quantify the relative abundance of major trophic groups. Differential fungal taxa associated with treatments were identified using LEfSe (linear discriminant analysis effect size), with the LDA threshold adjusted as required. Relationships between fungal community structure, soil physicochemical variables, and plant traits were examined using Spearman correlation and Mantel tests.

### 2.9 Statistical analyses

All data are presented as mean ± standard error (SE). Prior to statistical analysis, data were tested for normality using the Shapiro–Wilk test and for homogeneity of variances using Levene’s test. When assumptions of normality or homoscedasticity were not met, log- or square-root transformations were applied as appropriate before conducting parametric analyses.

A two-way analysis of variance (ANOVA) was performed to evaluate the main effects of AMF inoculation (CK, NM, GM), phosphorus supply (P0, P1, P2), and their interaction (AMF × P) on plant growth parameters, organ-level P concentrations, root colonization rate, soil physicochemical properties, and diversity indices. When significant effects were detected, *post hoc* comparisons were conducted using Duncan’s multiple range test at P < 0.05.

For correlation analyses, data distribution characteristics and sample size were considered prior to selecting statistical tests. Spearman’s rank correlation was used for treatment-level associations due to limited sample size and potential deviations from normality. Correlation coefficients (ρ) and associated P values were calculated to assess relationships between AMF colonization, plant growth traits, soil properties, and fungal community variables.

All statistical analyses were conducted using SPSS 26.0 (IBM, USA) and R version 4.2.2. Figures were generated using Origin 2022 and the R package ggplot2.

## 3 Results

### 3.1 AMF colonization under different inoculation treatments

**Figure 1:**
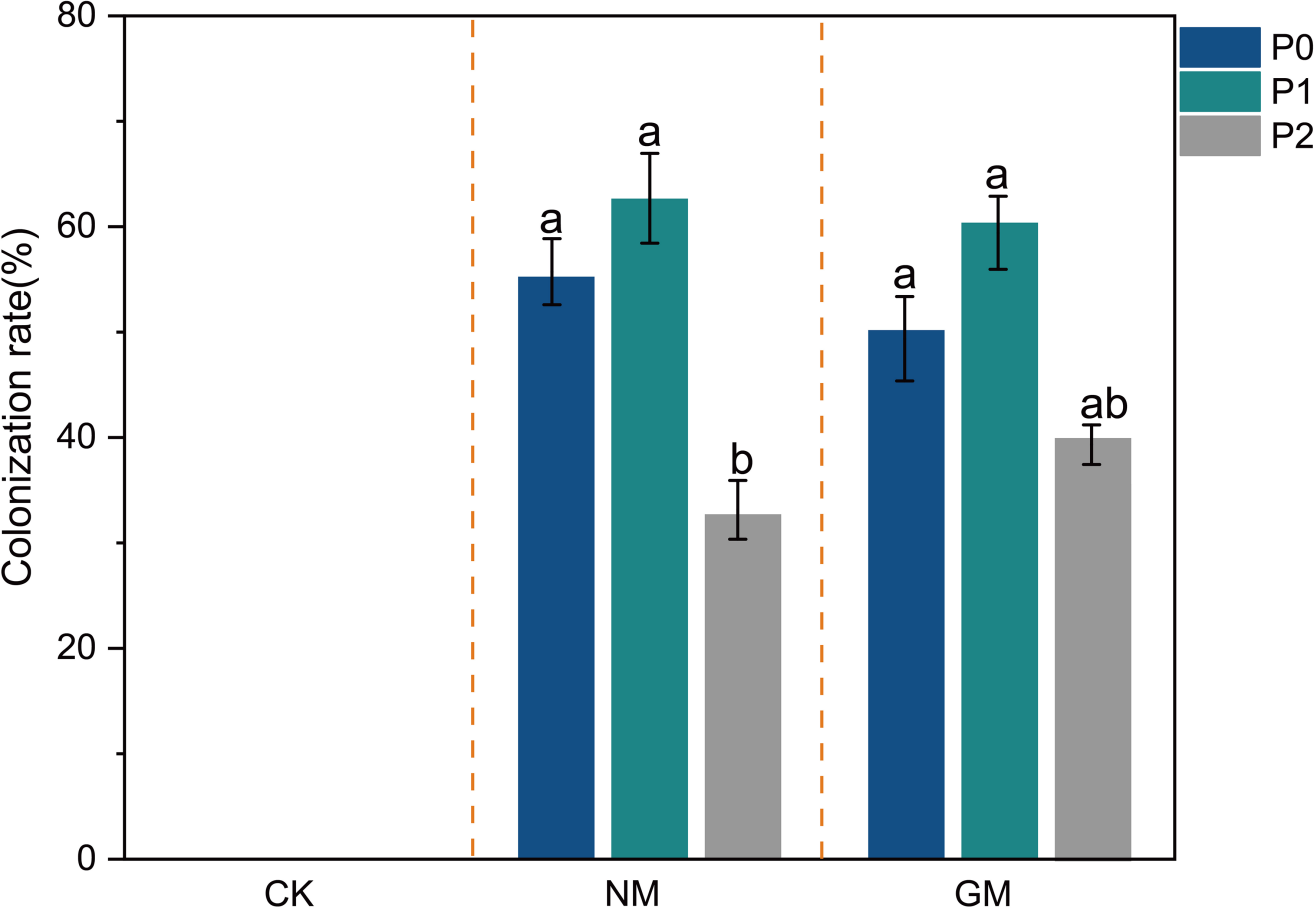
AMF colonization rate under different phosphorus supply and inoculation treatments. Different letters above bars indicate significant differences among phosphorus levels within each inoculation treatment (Duncan’s multiple range test, P < 0.05).

AMF colonization was assessed at harvest to verify the establishment of symbiosis under different inoculation and phosphorus treatments (**Figure 1**). In the CK treatment, no typical mycorrhizal structures were observed across all phosphorus levels, and colonization rate remained close to zero, confirming the effectiveness of soil sterilization.

Under AMF inoculation, both NM and GM treatments exhibited clear root colonization. In the NM treatment, colonization rates were relatively high under P0 (55.40%) and P1 (62.40%), with no significant difference between these two levels. However, colonization rate significantly declined under P2 (32.40%). A similar pattern was observed in the GM treatment, where colonization under P0 and P1 did not differ significantly, while high phosphorus supply (P2) significantly reduced colonization to approximately 39.80%. Overall, elevated phosphorus supply suppressed AMF colonization in both inoculation treatments, whereas no significant difference was detected between P0 and P1.

### 3.2 Effects of phosphorus supply and AMF inoculation on seedling growth and biomass allocation

**Figure 2:**
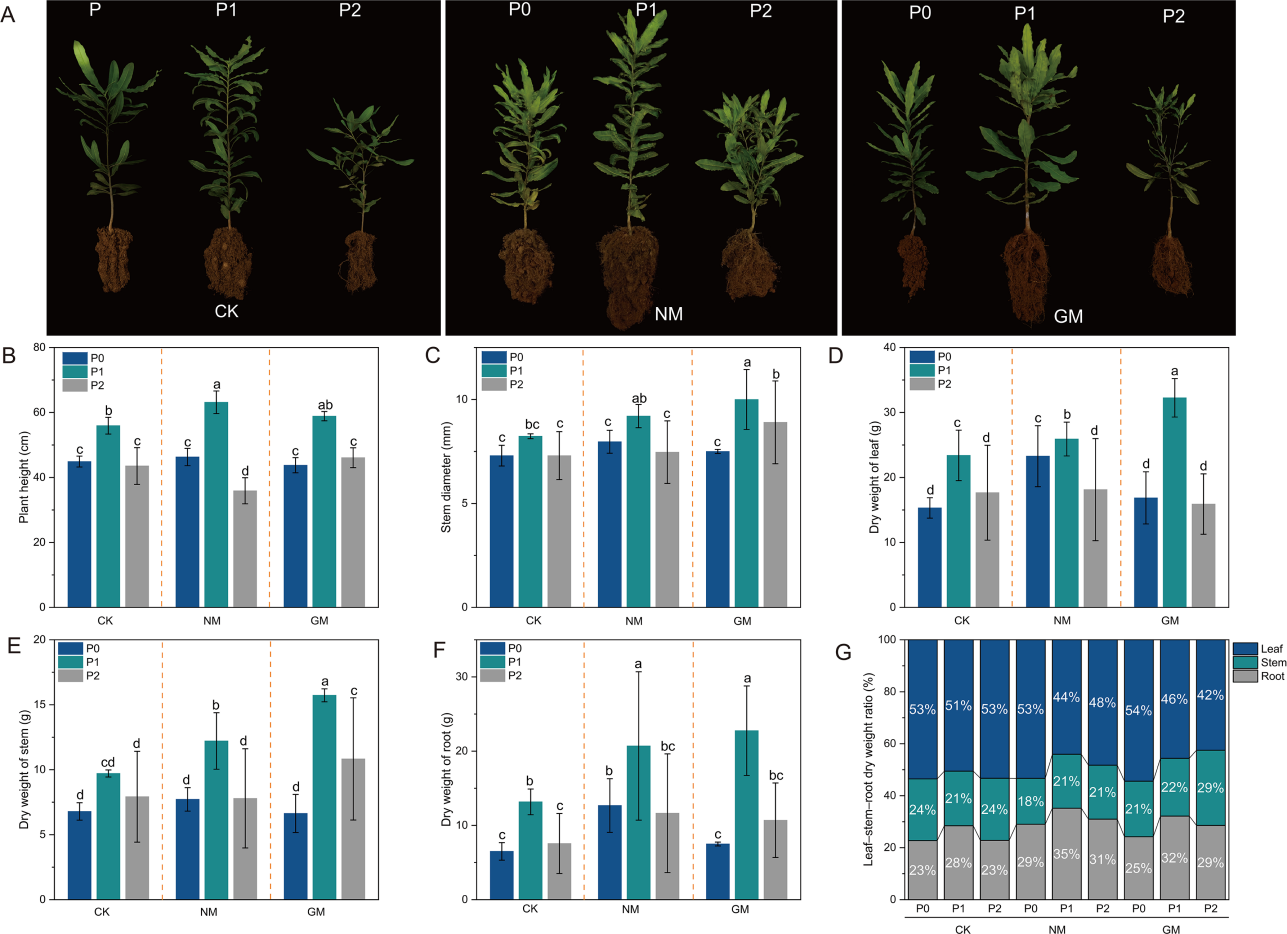
Effects of phosphorus supply and AMF inoculation on *Macadamia integrifolia* seedling growth and biomass. **(A)** Representative seedlings; **(B)** Plant height; **(C)** Stem diameter; **(D)** Leaf dry weight; **(E)** Stem dry weight; **(F)** Root dry weight; **(G)** Biomass allocation. Different lowercase letters indicate significant differences among P levels within each AMF treatment (Duncan, P < 0.05).

Phosphorus supply and AMF inoculation were associated with variation in seedling growth traits and organ-level biomass allocation in *Macadamia integrifolia* seedlings (**Figure 2**). Representative seedling morphology under different P and AMF treatments is shown in **Figure 2A**. Across inoculation treatments, plant height tended to be higher under P1 compared with P0 and P2 (**Figure 2B**). Within the NM treatment, plant height at P0 was higher than at P2, whereas no significant difference between P0 and P2 was detected in the GM and CK treatments. Stem diameter exhibited a broadly similar response, with values generally peaking at P1 (**Figure 2C**). No significant differences among P levels were detected in the CK treatment. By contrast, in the NM treatment, stem diameter was significantly greater at P1 than at P0 and P2, whereas in the GM treatment a clear gradation was observed, with stem diameter decreasing sequentially from P1 to P0 and then to P2.

Leaf, stem, and root biomass varied with phosphorus supply across inoculation treatments (**Figures 2D–F**). For leaf biomass, P1 resulted in significantly higher dry weight than P0 and P2 in both the CK and GM treatments, with no significant difference between P0 and P2, whereas in the NM treatment leaf biomass differed significantly among all three P levels, decreasing from P1 to P0 and then to P2. Stem biomass showed no significant differences among P levels in the CK treatment but was significantly greater at P1 than at P0 and P2 in the NM treatment; in the GM treatment, stem biomass differed significantly among P levels, with values highest at P1, followed by P2 and then P0. Root biomass differed less markedly among P levels: in all treatments, root dry weight at P1 was significantly higher than at P0, while P2 showed intermediate values and did not differ significantly from either P0 or P1.

Across all treatments, aboveground organs (leaf + stem) accounted for approximately 65–77% of total dry matter, while roots contributed 23–35% (**Figure 2G**). In the CK treatment, the proportion of aboveground biomass was higher at P0 and P2 than at P1, with a corresponding increase in the root fraction at P1. A similar shift in biomass partitioning was observed in the NM treatment, where the aboveground fraction declined and the root fraction increased at P1 relative to P0. In the GM treatment, the aboveground fraction was highest at P0, decreased at P1, and partially recovered at P2, with inverse changes in the root fraction across P levels. Effects of phosphorus supply and AMF inoculation on organ-level P concentration and P partitioning.

### 3.3 Effects of phosphorus supply and AMF inoculation on organ-level P concentration and P partitioning

**Figure 3:**
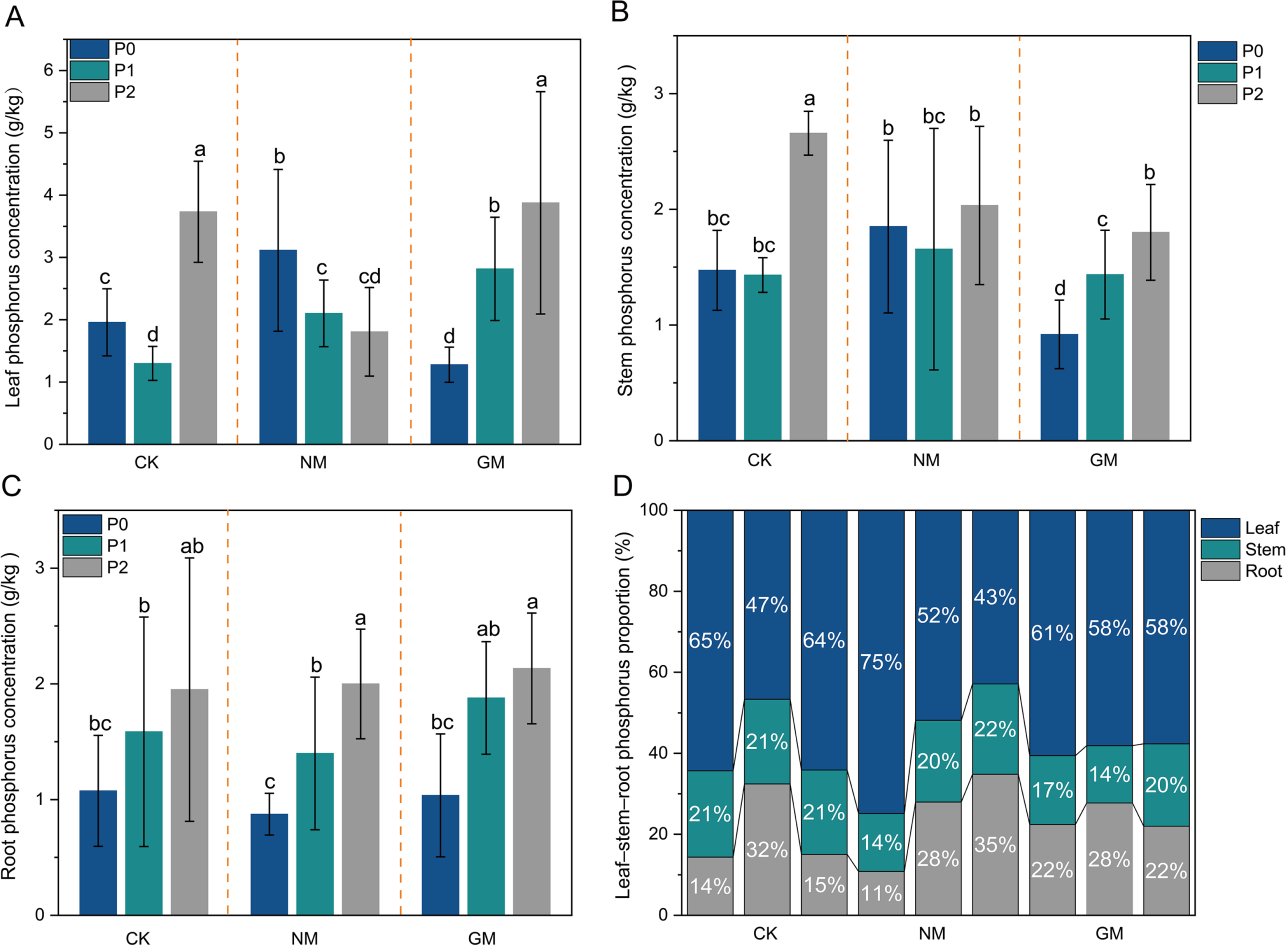
Organ-level phosphorus concentrations and whole-plant P partitioning under different phosphorus supply and AMF inoculation. **(A)** Leaf P concentration; **(B)** Stem P concentration; **(C)** Root P concentration; **(D)** Proportional P partitioning among organs. Different lowercase letters indicate significant differences among P levels within each AMF treatment (Duncan, P < 0.05).

The organ-level phosphorus (P) concentrations varied across P supply levels and AMF inoculation treatments, with distinct response patterns among plant organs (**Figure 3**). Leaf P concentration showed pronounced variation among treatments (**Figure 3A**). In the CK treatment, leaf P concentration tended to be higher at P2 than at P0 and P1. In the NM treatment, leaf P concentration at P0 was higher than at P2, whereas P1 did not differ significantly from either level. In the GM treatment, leaf P concentration was highest at P2, while no significant difference was detected between P0 and P1. Differences in leaf P concentration among AMF treatments were treatment- and P-level dependent, with NM showing higher values than GM at P0, whereas under P2, CK and GM exhibited higher leaf P concentrations than NM.

Stem phosphorus (P) concentration exhibited comparatively modest and treatment-dependent variation across phosphorus levels (**Figure 3B**). In the CK treatment, stem P concentration was significantly higher at P2 than at P0 and P1, whereas no significant differences among P levels were detected in the NM treatment. In the GM treatment, stem P concentration displayed a consistent monotonic increase across phosphorus levels, following the order P2 > P1 > P0. Overall, the magnitude of variation in stem P concentration was smaller than that observed in leaf tissue. Root P concentration increased consistently with increasing P supply across all AMF treatments (**Figure 3C**). Root P concentration at P2 exceeded that at P0 and P1, whereas differences among AMF inoculation treatments were not significant, indicating a dominant effect of P supply on belowground P concentration.

Despite variation in organ-level P concentrations, proportional P partitioning among organs remained relatively stable across treatments (**Figure 3D**). Leaves consistently accounted for the largest proportion of total plant P (approximately 43–75%), followed by roots (11–35%) and stems (14–22%). Variation in P partitioning among AMF treatments and P levels was modest, suggesting conservative whole-plant P allocation under the experimental conditions.

### 3.4 Effects of phosphorus supply and AMF inoculation on soil available P and pH in rhizosphere and non-rhizosphere soils

Soil available phosphorus (P) and pH varied across P supply levels and between rhizosphere and non-rhizosphere compartments, with responses differing among AMF inoculation treatments (**Tables 1**, **2**). In non-rhizosphere soil, available P increased with increasing P addition across all inoculation treatments. CK and NM showed significantly higher P at P2 compared to P0 and P1, whereas GM increased steadily from P0 to P2. Two-way ANOVA indicated that both AMF inoculation and P supply significantly affected non-rhizosphere P, with a significant inoculation × P supply interaction (**Table 2**). In rhizosphere soil, available P also increased with P supply, and differences among treatments were more pronounced. Under P0, NM exhibited significantly higher rhizosphere P than GM, while under P2, GM had the highest P among all treatments. Here, P supply had a strong effect, and the interaction between inoculation and P supply was also significant, whereas inoculation alone was not significant.

**Table 1:** Soil available P and pH (mean ± SE) in non-rhizosphere and rhizosphere; letters indicate within-column differences.

Sample	Non-rhizosphere available P (mg kg^-^¹)	Rhizosphere available P (mg kg^-^¹)	Non-rhizosphere pH	Rhizosphere pH
CK-P0	1.78 ± 0.29 d	1.59 ± 0.11 d	4.97 ± 0.03 a	4.94 ± 0.10 b
CK-P1	4.06 ± 0.72 cd	3.94 ± 0.82 cd	5.05 ± 0.03 a	5.07 ± 0.13 b
CK-P2	19.42 ± 1.11 b	20.11 ± 1.24 b	4.84 ± 0.01 a	4.97 ± 0.05 b
NM-P0	4.06 ± 0.67 cd	7.30 ± 0.98 c	4.91 ± 0.05 a	5.00 ± 0.27 b
NM-P1	5.33 ± 0.95 c	6.79 ± 0.47 c	4.90 ± 0.06 a	5.06 ± 0.13 b
NM-P2	20.56 ± 0.39 b	22.40 ± 4.63 b	4.80 ± 0.03 a	4.94 ± 0.04 b
GM-P0	1.08 ± 0.19 d	1.40 ± 0.21 d	5.04 ± 0.10 a	6.07 ± 0.92 a
GM-P1	5.20 ± 1.04 c	4.95 ± 0.79 cd	4.82 ± 0.05 a	5.34 ± 0.17 b
GM-P2	23.92 ± 1.31 a	33.44 ± 1.33 a	4.88 ± 0.08 a	4.99 ± 0.09 b

Different lowercase letters indicate significant differences among treatments (P < 0.05) based on Tukey’s HSD *post-hoc* test. For the complete two-way ANOVA results (main effects and interaction), see **Table 2**.

**Table 2:** Two-way ANOVA results for non-rhizosphere and rhizosphere soil P and pH.

Parameter	Source	Df	F value	P value	Significance
Non-rhizosphere P	Inoculation (I)	2	7.235	0.0049	**
	Phosphorus (P)	2	175.492	<0.001	***
	I × P	4	9.08	0.003	***
Rhizosphere P	Inoculation (I)	2	2.999	0.0751	NS
	Phosphorus (P)	2	369.111	<0.001	***
	I × P	4	3.369	0.0317	*
Non-rhizosphere pH	Inoculation (I)	2	4.928	0.0196	*
	Phosphorus (P)	2	13.762	0.0002	***
	I × P	4	7.514	0.000962	***
Rhizosphere pH	Inoculation (I)	2	5.672	0.0123	*
	Phosphorus (P)	2	2.648	0.098	NS
	I × P	4	2.781	0.0584	NS

**Significance: NS, not significant, *P < 0.05, **P < 0.01, ***P < 0.001.

For soil pH, non-rhizosphere pH was influenced by both AMF inoculation and P supply, with a significant interaction effect. In rhizosphere soil, pH was mainly affected by AMF inoculation. These results suggest that non-rhizosphere pH responds to the combined effects of P supply and inoculation, whereas rhizosphere pH is less sensitive to P supply. Soil available phosphorus (P) and pH varied across P supply levels and between rhizosphere and non-rhizosphere compartments, with responses differing among AMF inoculation treatments (**Tables 1**, **2**).

### 3.5 Effects of P supply and AMF inoculation on rhizosphere fungal α-diversity and community composition

**Figure 4:**
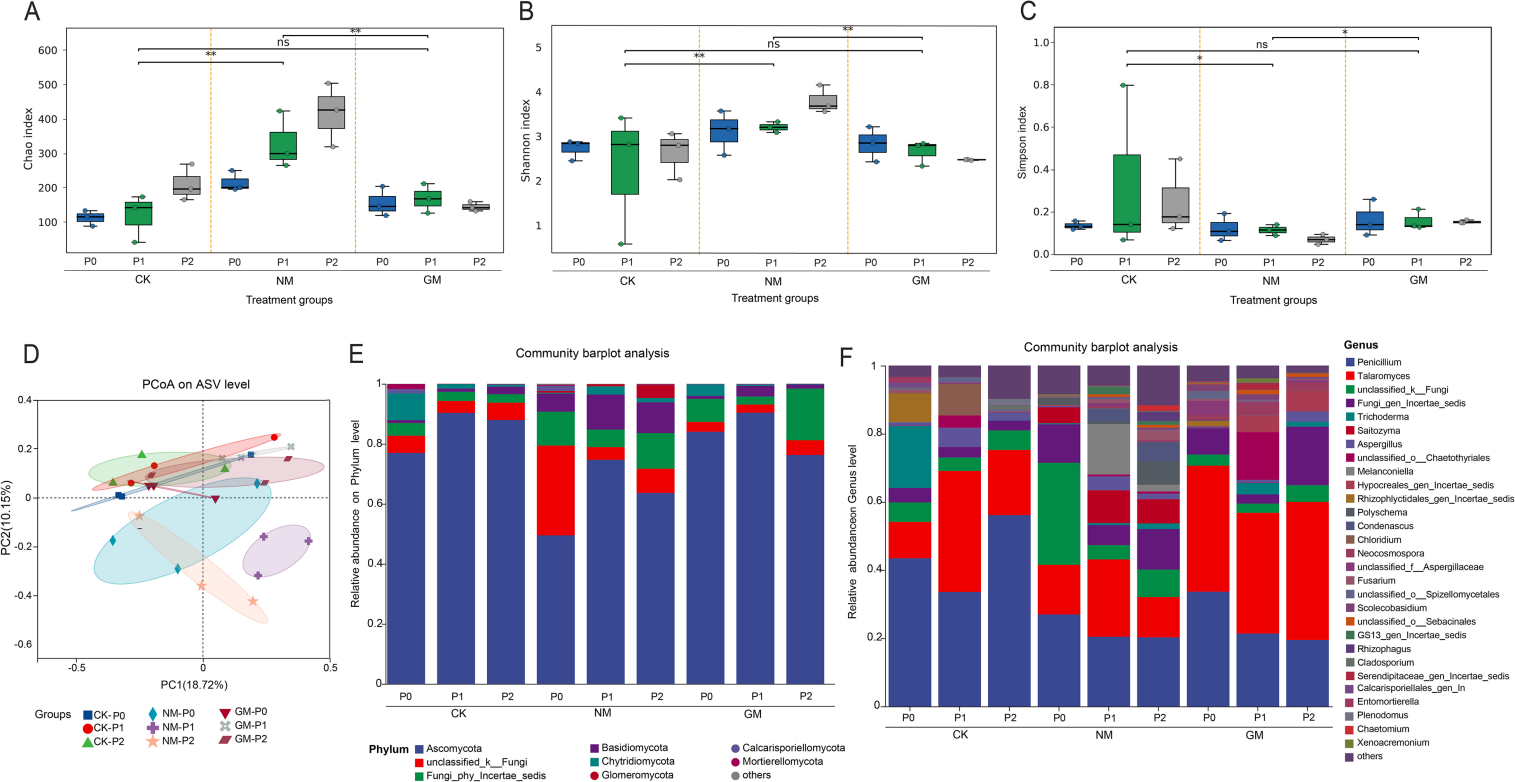
Rhizosphere fungal α-diversity and community composition under different phosphorus supply and AMF inoculation.**(A–C)** α-diversity indices (Chao1, Shannon, Simpson) at the ASV level; **(D)** PCoA based on Bray–Curtis distances (percent variance explained by PC1 and PC2 indicated); **(E)** Taxonomic composition at the phylum level; **(F)** Taxonomic composition at the genus level.

Rhizosphere fungal α-diversity varied across phosphorus (P) supply levels and AMF inoculation treatments (**Figure 4**). Chao1 richness differed among treatments, with higher values generally observed in AMF-inoculated treatments compared with CK, particularly under high P supply (**Figure 4A**). Shannon diversity also showed treatment-dependent variation, with the highest values observed in the GM treatment at P2, whereas CK exhibited lower diversity across P levels (**Figure 4B**). Simpson indices displayed patterns broadly consistent with those of Shannon diversity, although differences among P levels were not consistent across inoculation treatments (**Figure 4C**). The detailed α-diversity indices for each sample are provided in **Supplementary Table S2**.

Principal coordinates analysis (PCoA) based on Bray–Curtis dissimilarities indicated differences in rhizosphere fungal community composition among inoculation treatments and P levels (**Figure 4D**). Samples from CK and AMF-inoculated treatments showed partial separation, with additional variation observed between NM and GM treatments, suggesting treatment-associated differences in community composition.

At the phylum level, Ascomycota dominated (50–80%), followed by Basidiomycota, while Chytridiomycota, Mortierellomycota, and Calcarisporiellomycota occurred at low relative abundances (**Figure 4E**). AMF inoculation substantially increased the relative abundance of Glomeromycota, with the greatest increases observed in GM. At the genus level (**Figure 4F**), compositional changes were more pronounced: *Penicillium* and *Talaromyces* were enriched in CK and NM but decreased in GM, whereas *Rhizophagus* increased significantly under AMF inoculation. *Trichoderma* and *Aspergillus* showed P-related variation, with abundances fluctuating across P levels. Taken together, rhizosphere fungal α-diversity and community composition exhibited treatment-associated variation across P supply levels and AMF inoculation treatments, with differences evident at both the diversity and taxonomic composition levels.

### 3.6 Figures effects of P supply and AMF inoculation on differential fungal taxa based identified by LefSe

**Figure 5:**
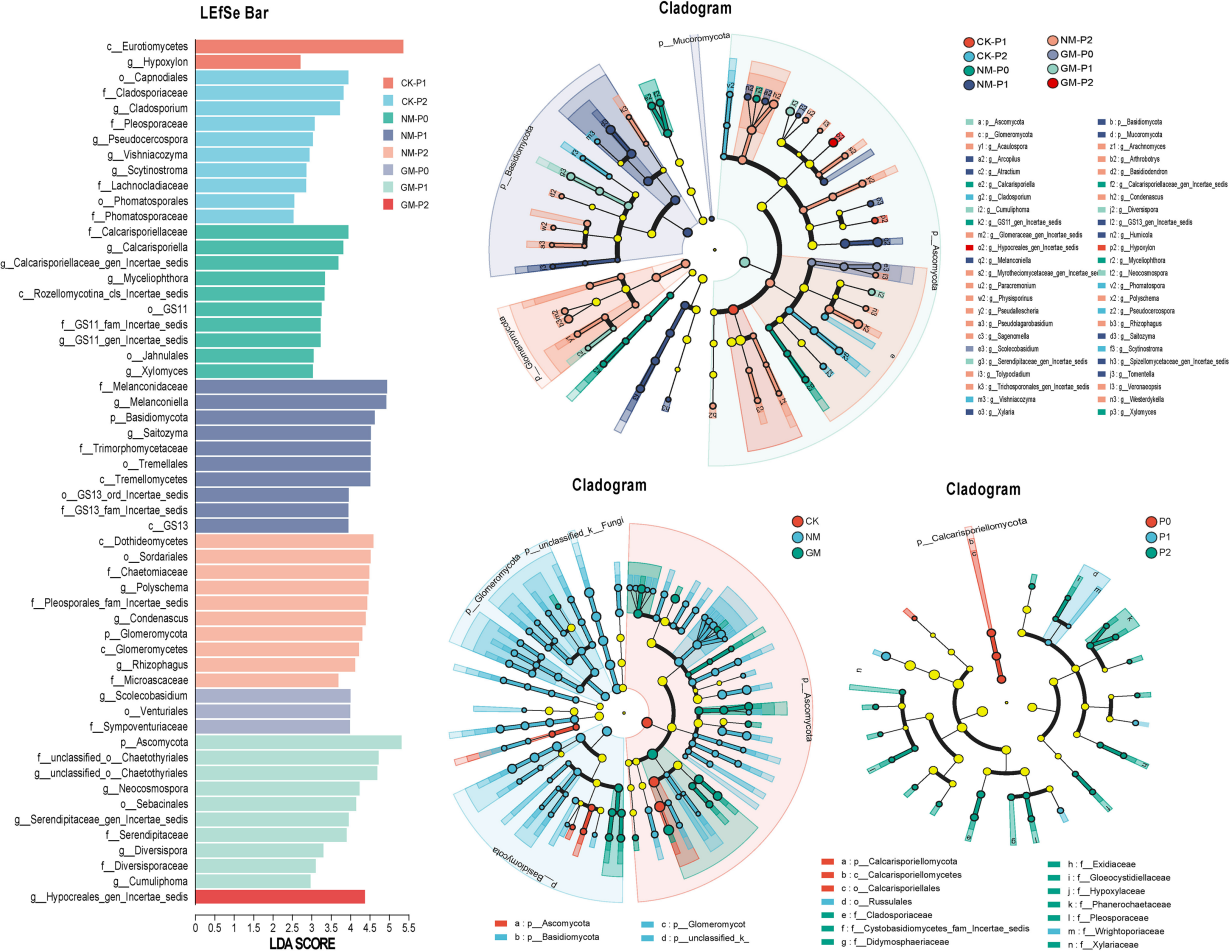
Differentially associated fungal taxa identified by LEfSe under different phosphorus supply and AMF inoculation.Fungal lineages significantly associated with specific treatment combinations at each P level (P0, P1, P2) were identified by LEfSe (LDA ≥ 2.0, P < 0.05). Taxa are shown at different taxonomic ranks.

Linear discriminant analysis effect size (LEfSe) analysis identified fungal taxa that were differentially associated with combinations of phosphorus (P) supply and AMF inoculation treatments (LDA ≥ 2.0, *p* < 0.05; **Figure 5**). In the CK treatment, relatively few taxa were identified as discriminatory across P levels. Under P1, *Eurotiomycetes* was the primary taxon associated with CK, whereas under P2, several *Ascomycota* lineages, including *Sordariomycetes* and *Dothideomycetes*, as well as multiple *Basidiomycota* groups, were identified.

In the NM treatment, a greater number of discriminatory taxa were detected across P levels. Under P0, taxa affiliated with *Rozellomycota* and *Ascomycota* were identified. Under P1, additional *Basidiomycota*-associated lineages were detected. Under P2, discriminatory taxa included representatives of *Glomeromycota* (e.g. *Rhizophagus* and *Diversispora*), as well as several *Ascomycota* groups, including *Hypocreales*, *Microascales*, *Myrotheciomycetaceae*, and *Pseudallescheria*. In the GM treatment, fewer discriminatory taxa were detected across P levels. Under P0, *Scolecobasidium* was identified, whereas under P1, taxa including *Diversispora*, *Neocosmospora*, and *Cumuliphoma* were detected. Under P2, a single unclassified *Hypocreales* lineage was identified.

Collectively, LEfSe analysis revealed treatment-associated differences in the taxonomic composition of the rhizosphere fungal community across P supply levels and AMF inoculation treatments, with the identity of discriminatory taxa varying among treatments.

### 3.7 Effects of phosphorus supply and AMF inoculation on the composition of rhizosphere fungal functional guilds

**Figure 6:**
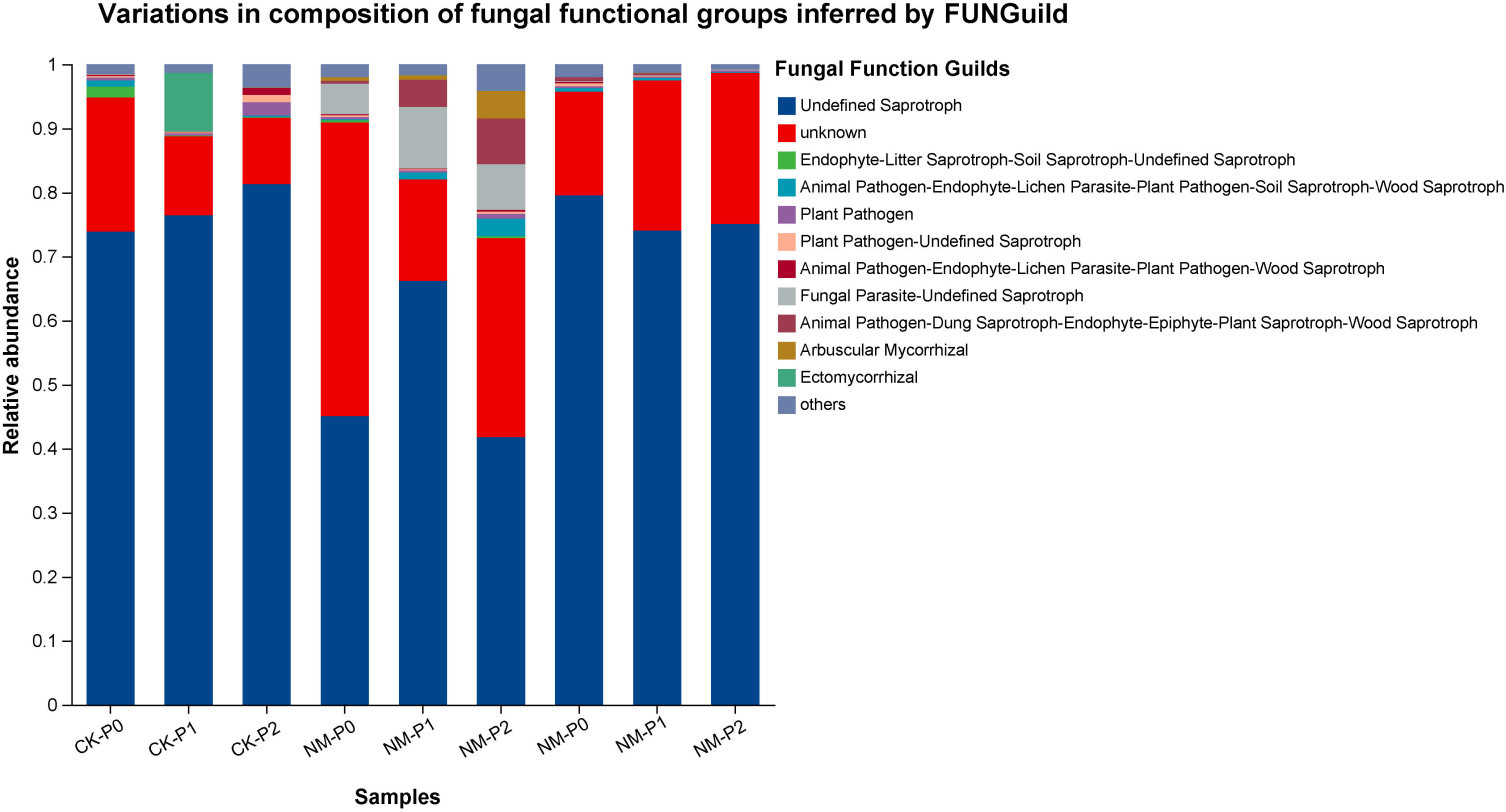
Relative abundance of fungal functional guilds inferred by FUNGuild in the rhizosphere.

Fungal functional guilds inferred using FUNGuild indicated that the rhizosphere community was primarily composed of taxa assigned to saprotrophic, mycorrhizal, pathogen/parasite, and undefined guilds, with their relative proportions varying among phosphorus (P) levels and AMF inoculation treatments (**Figure 6**). In CK, taxa assigned to the saprotrophic guild dominated the rhizosphere fungal community across all P levels, accounting for more than 75% of relative abundance, with higher proportions observed at P2 compared with P0 and P1. Taxa assigned to the mycorrhizal guild were detected at low proportions across P levels, with slightly higher values at P1. The relative abundance of taxa assigned to pathogen/parasite guilds remained low, showing a modest increase at P2.

In the NM treatment, taxa assigned to the mycorrhizal guild accounted for a greater proportion of the community at P0 and P2 compared with CK, whereas taxa assigned to saprotrophic guilds showed lower relative abundance, particularly at P2. Taxa assigned to pathogen/parasite guilds remained lower than those observed in CK across all P levels. In the GM treatment, taxa assigned to the mycorrhizal guild were also present at higher relative abundance than in CK, with the highest proportions observed at P0, followed by a decline toward P2. Saprotrophic guilds remained the dominant component of the community but occurred at lower relative abundance than in CK and generally higher abundance than in NM. Several composite guilds, including endophyte–plant pathogen and fungal parasite groups, were detected at higher proportions compared with CK. As a whole, the relative composition of fungal functional guilds in the rhizosphere differed among P levels and AMF inoculation treatments, with variation observed in the proportional contributions of saprotrophic, mycorrhizal, and pathogen/parasite-associated taxa. It should be noted that FUNGuild-based assignments represent functional inference at the taxonomic level rather than direct measurement of metabolic activity. Therefore, interpretations regarding phosphorus mineralization or nutrient cycling should be considered indicative rather than definitive.

### 3.8 Correlations between fungal taxa and plant growth traits and P-related variables

**Figure 7:**
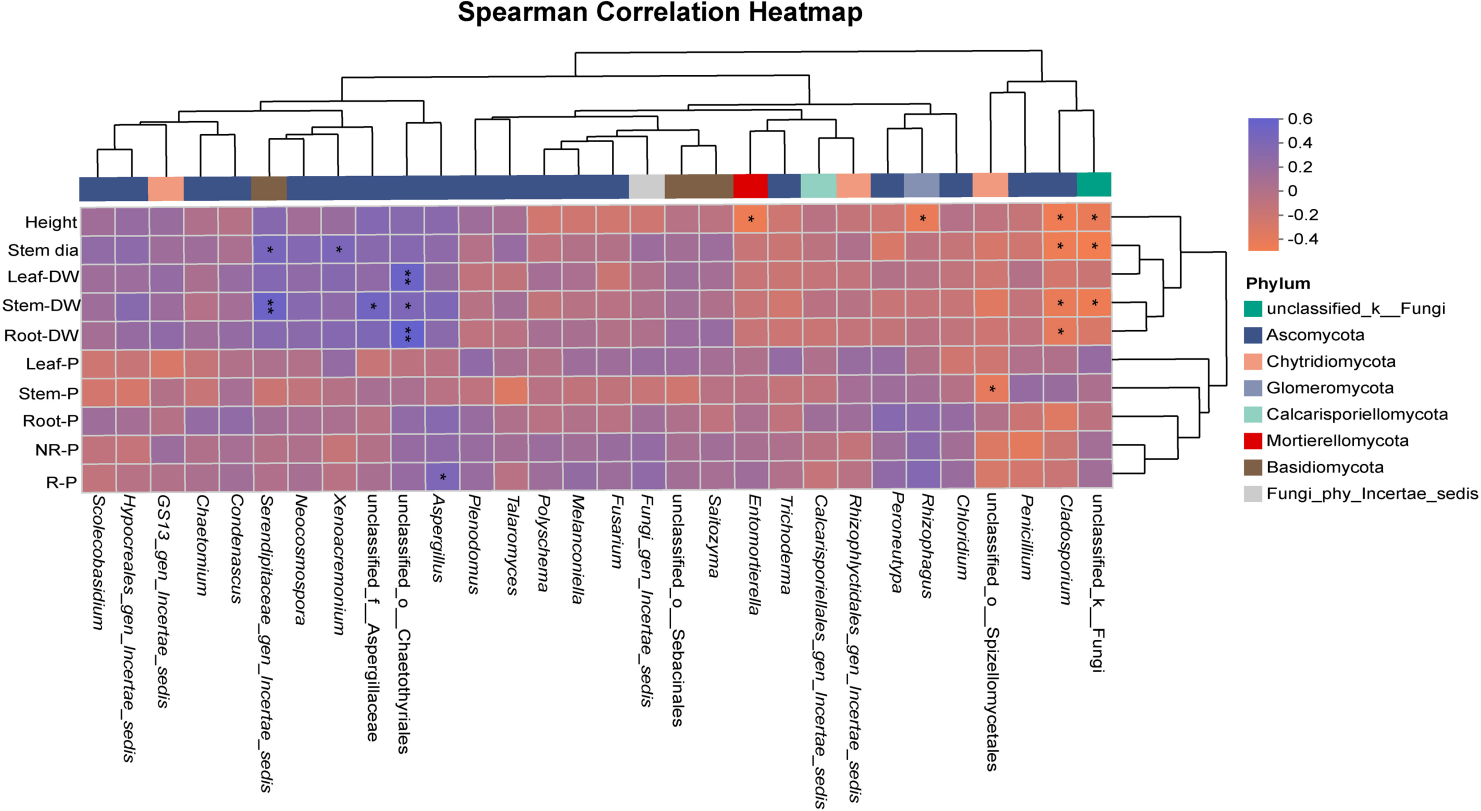
Spearman correlation heatmap between fungal taxa and plant growth and phosphorus-related variables. Significance levels are indicated as: *p<0.05, ** p<0.01.

Spearman correlation analysis revealed associations between the relative abundance of selected fungal taxa and plant growth traits as well as phosphorus (P)-related variables (**Figure 7**). Several taxa showed positive correlations with aboveground growth traits. For example, the relative abundance of unclassified_k:Fungi was positively correlated with plant height, stem diameter, and stem dry weight, whereas *Cladosporium* showed positive correlations with plant height, stem diameter, stem dry weight, and root dry weight. *Rhizophagus* and *Entomortierella* each exhibited positive correlations with plant height. Associations between fungal taxa and soil or tissue P-related variables were also observed. The relative abundance of *Aspergillus* was positively correlated with rhizosphere available P. Unclassified_o:Chaetothyriales showed positive correlations with leaf dry weight and root dry weight, as well as a positive association with stem dry weight. In addition, Serendipitaceae_gen_Incertae_sedis exhibited positive correlations with stem diameter and stem dry weight. Taken as a whole, correlation analysis indicated that the relative abundance of different fungal taxa was variably associated with plant growth traits and P-related variables, highlighting heterogeneous association patterns across taxa.

### 3.9 Correlations among AMF colonization, phosphorus uptake efficiency, and plant growth

**Figure 8:**
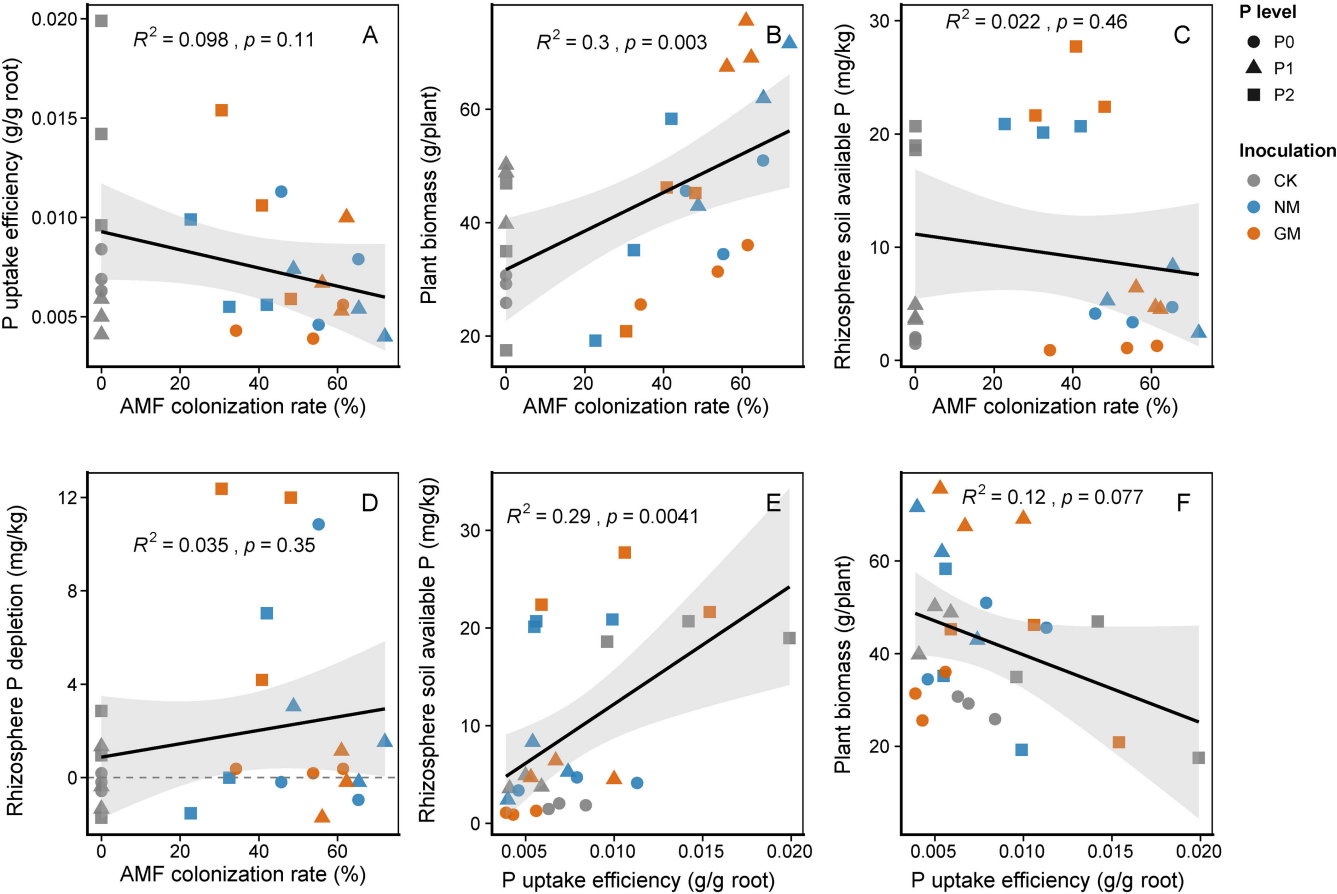
**(A)** AMF colonization rate vs. phosphorus uptake efficiency, **(B)** AMF colonization rate vs. total plant biomass, **(C)** AMF colonization rate vs. rhizosphere available P, **(D)** AMF colonization rate vs. rhizosphere P depletion, **(E)** Phosphorus uptake efficiency vs. rhizosphere available P, **(F)** Phosphorus uptake efficiency vs. total plant biomass.

AMF colonization rate was highly positively correlated with total plant biomass (**Figure 8B**; R² = 0.30, P = 0.003), indicating that plants with higher levels of root colonization tended to achieve greater overall growth. In contrast, no significant relationship was detected between AMF colonization and phosphorus (P) uptake efficiency (**Figure 8A**; R² = 0.098, P = 0.11), nor with rhizosphere available P content (**Figure 8C**; R² = 0.022, P = 0.46) or rhizosphere P depletion (**Figure 8D**; R² = 0.035, P = 0.35). These results suggest that while AMF colonization strongly supports plant growth potential, it does not directly translate into enhanced P uptake efficiency or immediate changes in rhizosphere P status.

Further analysis revealed a highly significant negative correlation between P uptake efficiency and rhizosphere available P content (**Figure 8E**; R² = 0.29, P = 0.0041), indicating that plants with higher P acquisition capacity tended to deplete soil P more effectively. By contrast, P uptake efficiency was not significantly correlated with total plant biomass (**Figure 8F**; R² = 0.12, P = 0.077), suggesting that enhanced P uptake does not necessarily result in larger plant size, likely reflecting trade-offs in resource allocation and growth strategies. These findings highlight a degree of functional decoupling between AMF colonization, P acquisition, and plant growth, with each parameter contributing differently to plant performance and rhizosphere P dynamics.

## 4 Discussion

### 4.1 Biomass allocation responses to phosphorus supply under contrasting AMF inoculation treatments

Macadamia seedlings exhibited a unimodal biomass response along the external phosphorus (P) gradient, with maximal growth observed at the intermediate P level (P1). Reduced growth under low P (P0) likely reflects insufficient phosphorus to sustain optimal carbon assimilation, whereas growth suppression at high P (P2) may result from nutrient imbalances or altered soil chemistry, such as Ca–P precipitation, constraining biomass accumulation (Bechtaoui et al., 2021; Carstensen et al., 2018). These observations suggest that biomass accumulation is governed by the coordination between carbon acquisition and phosphorus availability rather than by a simple linear fertilization response (Zhao et al., 2020).

Importantly, the significant interaction between phosphorus supply and AMF inoculation indicates that AMF effects were strongly dependent on nutrient background, reflecting a strain functional traits–environmental adaptability matching pattern. Under low P, the indigenous AMF consortium maintained higher colonization and enhanced leaf P concentration, likely reflecting functional traits adapted to nutrient limitation, including high-affinity P transporters and extensive extraradical hyphal networks (Chiu and Paszkowski, 2019; G. Wang et al., 2023a).

Under moderate phosphorus supply (P1), *G. mosseae* exhibited stronger promotion of aboveground biomass accumulation compared with other treatments. This suggests that when phosphorus availability is not severely limiting, introduced AMF strains with high colonization vigor and rapid hyphal proliferation may enhance plant growth through improved carbon-phosphorus exchange dynamics (Wu et al., 2024). The higher colonization rates maintained by *G. mosseae* under P1 (Section 3.1) support this interpretation, indicating that its functional strategy prioritizes symbiotic establishment and resource delivery even when P is moderately available. Such context-dependent performance differences highlight functional differentiation between indigenous consortia and single-species inocula (Chen et al., 2017).

In contrast, under excessive phosphorus supply (P2), colonization intensity declined in both inoculation treatments, consistent with the well-documented suppression of AMF symbiosis under high P availability. Nevertheless, differences in plant biomass among inoculation treatments persisted, indicating that AMF-associated effects may involve both symbiotic and rhizosphere-mediated processes that extend beyond colonization intensity alone (Zhu et al., 2025). These findings underscore the context dependency of plant–AMF interactions and demonstrate that the functional outcomes of AMF inoculation shift across phosphorus gradients.

Across all treatments, 44-week-old seedlings maintained a consistent hierarchy of biomass allocation (leaf > root > stem), with roots exhibiting less variation than aboveground tissues (Poorter et al., 2012; Pregitzer, 2003). Root biomass exhibited less variation than aboveground tissues, suggesting that baseline nutrient acquisition capacity was maintained across P conditions (Marschner, 2012). Overall, these results emphasize that both phosphorus supply and AMF identity shape biomass allocation patterns through nutrient-context-dependent mechanisms (Wu et al., 2024). Collectively, these results emphasize that phosphorus availability and AMF identity jointly shape biomass allocation through nutrient-context-dependent mechanisms. Integrating strain-level functional traits (e.g., hyphal elongation, phosphorus uptake kinetics, enzyme activity) with community-level analyses would further clarify AMF-specific contributions.

### 4.2 AMF-mediated regulation of rhizosphere phosphorus and coordination with plant intrinsic P-acquisition strategies

Building on these growth patterns, we examined how AMF inoculation and phosphorus supply interact to regulate rhizosphere P availability. Rhizosphere phosphorus did not increase linearly with external P supply but varied according to AMF identity and nutrient background (Wu et al., 2024). Under low P conditions (P0), seedlings inoculated with the indigenous AMF consortium exhibited higher rhizosphere available P and enhanced leaf P concentration compared with non-inoculated treatments, suggesting functional traits optimized for nutrient-limited environments, potentially involving high-affinity phosphate transporters or extensive hyphal network exploration (Liu et al., 2025). Under low P conditions (P0), seedlings inoculated with the indigenous AMF consortium were associated with relatively higher rhizosphere available P compared with non-inoculated treatments, whereas under high P (P2), seedlings inoculated with *Glomus mosseae* showed higher plant P accumulation. These contrasting patterns suggest that the effects of AMF inoculation on P-related responses are context-dependent, varying between nutrient-limited and nutrient-sufficient environments (Tibbett et al., 2022). Importantly, these observations reflect treatment-level patterns at the rhizosphere scale and should not be interpreted as direct evidence of enhanced P uptake or translocation by AMF (Lu et al., 2023).

Under excessive phosphorus supply (P2), increased soil available P did not correspond to proportional increases in plant growth. This may partly reflect altered soil ionic chemistry, such as Ca–P precipitation or nutrient imbalances, which can influence P speciation and availability in subtropical soils (Penn and Camberato, 2019). Such changes may modify rhizosphere processes and microbial interactions, while the observed decline in AMF colonization intensity under high P further supports the well-documented downregulation of AMF symbiosis under nutrient-rich conditions (Lin et al., 2020).

In addition to AMF-mediated pathways, macadamia (*Macadamia integrifolia*), as a member of the Proteaceae, possesses intrinsic P-acquisition strategies, including cluster root formation and organic acid exudation (Vance et al., 2003). The coexistence of these autonomous strategies with AMF symbiosis raises the possibility of functional complementarity or trade-offs across P gradients. Under low P, indigenous AMF may complement cluster root-mediated mobilization by accessing distinct soil P pools, whereas under moderate P, growth promotion by *G. mosseae* may reflect shifts in carbon allocation when AMF provides adequate P (Fang et al., 2024). Although cluster root formation and rhizosphere exudation were not directly quantified in this study, context-dependent shifts in colonization intensity and plant performance suggest that phosphorus-acquisition strategies in macadamia are dynamically regulated.

Moreover, while autoclaving effectively eliminated viable AMF propagules, residual microbial DNA or dormant structures (e.g., bacterial endospores, heat-tolerant fungal spores) may persist. These residuals are expected to be uniform across treatments but should be considered when interpreting rhizosphere microbial responses, particularly regarding AMF colonization and community composition. These results indicate that rhizosphere P dynamics in macadamia seedlings are jointly shaped by nutrient background and AMF identity, and that plant–microbe interactions operate within a coordinated framework of intrinsic and symbiotic phosphorus-acquisition strategies. Future studies integrating cluster root development (Neumann et al., 2000), organic acid secretion (Sas et al., 2001), and isotopic P tracing (Cruz-Paredes and Gavito, 2020) will provide deeper insight into the regulatory mechanisms underlying these complementary pathways.

### 4.3 Context-dependent associations between phosphorus supply, AMF inoculation, and rhizosphere fungal diversity

Patterns of rhizosphere fungal diversity were not explained by phosphorus (P) availability alone but varied with AMF inoculation treatment and nutrient background. Previous studies have reported that AMF inoculation can be associated with changes in fungal richness under specific nutrient conditions (Gao et al., 2021). In the present study, increases in α-diversity were more pronounced in the *Glomus mosseae* inoculation treatment under higher P supply, whereas the indigenous AMF consortium was associated with comparatively higher diversity under low P conditions. These patterns suggest that relationships between AMF inoculation and fungal diversity are context dependent and influenced by both inoculum identity and P availability, rather than following a single monotonic response to P supply (Wang et al., 2023b).

At the taxonomic level, Ascomycota and Basidiomycota dominated the rhizosphere fungal community across all treatments, while taxa affiliated with Glomeromycota increased in relative abundance in AMF-inoculated treatments. Reductions in the relative abundance of several saprotrophic genera under AMF inoculation may reflect shifts in resource availability or competitive interactions among fungal groups across P gradients (Han et al., 2025), although the specific processes underlying these patterns were not directly examined. Variation in the representation of multiple taxonomic and functional groups along the P gradient further indicates that nutrient background influenced community composition, while AMF inoculation modified how these compositional changes were expressed (Lekberg et al., 2021).

Analyses based on LEfSe and FUNGuild consistently distinguished AMF-inoculated treatments from non-inoculated controls, indicating that AMF inoculation was associated with changes in rhizosphere fungal community composition under contrasting P conditions (Xu et al., 2018). Together, these results suggest that AMF inoculation did not simply alter overall diversity metrics but was associated with shifts in the relative contributions of dominant and low-abundance fungal groups across nutrient contexts. These compositional patterns provide an important framework for interpreting downstream functional inferences, while emphasizing the need for caution when extrapolating from community structure to ecological function (Waring et al., 2022). Future work employing AMF-specific markers will help disentangle competitive dynamics between indigenous and introduced AMF strains. Direct quantification of functional genes (e.g., phoD) and enzyme activities would provide more robust mechanistic evidence linking community structure to phosphorus transformation processes (Khan et al., 2023; Lu et al., 2022).

### 4.4 Ecological associations of dominant fungal taxa under contrasting phosphorus and AMF treatments

Fungal taxa contributing to community compositional shifts did not respond uniformly across phosphorus (P) levels and AMF inoculation treatments, but instead showed distinct patterns of association with P availability and plant-related traits. Taxa such as *Rhizophagus* and *Entomortierella* increased in relative abundance under AMF inoculation, consistent with their previously reported ecological associations with mycorrhizal or root-associated environments (Lahrach et al., 2024). Other genera, including *Cladosporium*, were more abundant in treatments where aboveground biomass was relatively higher, suggesting potential links with plant-associated processes that have been reported in earlier studies (Jiang et al., 2021). In contrast, genera such as *Aspergillus* were more prevalent in treatments characterized by higher rhizosphere available P, in agreement with their documented roles in soil P mobilization and saprotrophic activity (Li et al., 2016). These observations indicate that fungal taxa differed in the environmental contexts in which they became more prominent and that AMF inoculation altered the expression of these patterns across P supply levels. Importantly, these interpretations are based on correlation and relative abundance patterns rather than direct measurements of functional activity, and further experimental work would be required to resolve the physiological mechanisms involved (Emmett et al., 2021). At the community level, responses to AMF inoculation therefore appear to reflect shifts in the relative contributions of multiple fungal groups rather than uniform changes in diversity alone (Chen et al., 2024; Gong et al., 2024).

Taken together, the combined influence of P supply and AMF inoculation was expressed not as a single dominant response trait, but as coordinated variation in biomass allocation, rhizosphere chemical properties, and fungal community composition. From this perspective, nutrient context defined the baseline conditions under which AMF-related associations emerged, while AMF inoculation modified how these conditions were reflected in community structure. These patterns underscore the importance of interpreting AMF effects as context-dependent associations within the plant–soil–microbe system rather than as direct functional drivers (Barber et al., 2013).

## 5 Conclusion

This study demonstrates that phosphorus supply and arbuscular mycorrhizal fungi (AMF) inoculation jointly shape the growth, biomass allocation, and rhizosphere fungal community structure of *Macadamia integrifolia* seedlings in a context-dependent manner. Seedling growth and organ-level phosphorus (P) concentration exhibited non-linear responses along the phosphorus gradient, with moderate P availability (P1, 40 mg·kg^-^¹) supporting maximal aboveground biomass. Specifically, under P1 conditions, *Glomus mosseae* inoculation increased aboveground biomass by approximately 42.9% and root P concentration by 18.4%, highlighting its potential as a preferred inoculation strategy in macadamia seedling production. The indigenous AMF consortium was more effective under low-P conditions, enhancing leaf P concentration and rhizosphere P availability. High phosphorus supply suppressed AMF colonization in both treatments, indicating downregulation of symbiosis under nutrient-rich conditions.

Rhizosphere fungal α-diversity and community composition were influenced by both P availability and AMF identity. AMF inoculation increased Glomeromycota abundance and shifted the balance of saprotrophic, mycorrhizal, and pathogen-associated guilds. The relative abundance of dominant taxa correlated with plant growth traits and P-related variables, while AMF colonization correlated strongly with total plant biomass but not short-term P uptake efficiency, highlighting functional differentiation between growth promotion and immediate nutrient acquisition.

These findings provide quantitative guidance for practical applications: under moderate P conditions, *G. mosseae* can be recommended to maximize seedling growth and root P accumulation, whereas local AMF consortia are preferable in low-P soils. However, limitations exist: this pot experiment cannot fully replicate field soil heterogeneity, and long-term cropping effects were not considered. Future studies should explore AMF inoculation combined with phosphorus and potassium fertilization, construct multi-strain inoculation systems, and incorporate direct measurements of cluster root activity, organic acid exudation, and isotope-based P tracing to further elucidate plant–microbe interactions and optimize phosphorus-use efficiency in field settings.

## Data Availability

The original contributions presented in the study are included in the article/**Supplementary Material**. Further inquiries can be directed to the corresponding author.
